# Incidence and risk predictors of acute kidney injury among HIV-positive patients presenting with sepsis in a low resource setting

**DOI:** 10.1186/s12882-021-02451-6

**Published:** 2021-06-29

**Authors:** Davis Kimweri, Julian Ategeka, Faustine Ceasor, Winnie Muyindike, Edwin Nuwagira, Rose Muhindo

**Affiliations:** 1 Department of Internal Medicine, Mbarara University of Science and Technology, P.O BOX 1410, Mbarara, Uganda; 2 Immunosuppression Clinic, Mbarara Regional Referral Hospital, Mbarara, Uganda

**Keywords:** AKI, Sepsis, HIV, Uganda, Sub-Saharan Africa

## Abstract

**Background:**

Acute kidney injury (AKI) is a frequently encountered clinical condition in critically ill patients and is associated with increased morbidity and mortality. In our resource-limited setting (RLS), the most common cause of AKI is sepsis and volume depletion. Sepsis alone, accounts for up to 62 % of the AKI cases in HIV-positive patients.

**Objective:**

The major goal of this study was to determine the incidence and risk predictors of AKI among HIV-infected patients admitted with sepsis at a tertiary hospital in Uganda.

**Methods:**

In a prospective cohort study, we enrolled adult patients presenting with sepsis at Mbarara Regional Referral Hospital (MRRH) in southwestern Uganda between March and July 2020. Sepsis was determined using the qSOFA criteria. Patients presenting with CKD or AKI were excluded. Sociodemographic characteristics, physical examination findings, and baseline laboratory values were recorded in a data collection tool. The serum creatinine and urea were done at admission (0-hour) and at the 48-hour mark to determine the presence of AKI. We performed crude and multivariable binomial regression to establish the factors that predicted developing AKI in the first 48 h of admission. Variables with a p < 0.01 in the adjusted analysis were considered as significant predictors of AKI.

**Results:**

Out of 384 patients screened, 73 (19 %) met our inclusion criteria. Their median age was 38 (IQR 29–46) years and 44 (60.3 %) were male. The median CD4 T-cell count was 67 (IQR 35–200) cells, median MUAC was 23 (IQR 21–27) cm and 54 (74.0 %) participants were on a regimen containing Tenofovir Disoproxil Fumarate (TDF). The incidence of AKI in 48 h was 19.2 % and in the adjusted analysis, thrombocytopenia (Platelet count < 150) (adjusted risk ratio 8.21: 95 % CI: 2.0–33.8, *p* = 0.004) was an independent predictor of AKI.

**Conclusions:**

There is a high incidence of AKI among HIV-positive patients admitted with sepsis in Uganda. Thrombocytopenia at admission may be a significant risk factor for developing AKI. The association of thrombocytopenia in sepsis and AKI needs to be investigated.

## Background

Acute kidney injury (AKI) is a frequently encountered clinical condition in critically ill patients and imposes a heavy burden of illness in terms of morbidity and mortality. The global burden of AKI is approximately 13.3 million per year, with an estimated 85 % from developing countries[1]. In Uganda, the prevalence of AKI among patients with sepsis is about 16.3 % with a high in-hospital mortality of up to 21 % [1]. In resource-limited settings (RLS), the most common causes of AKI include sepsis and volume depletion which account for 43 and 17 % respectively in HIV-negative patients and up to 62 and 20 % respectively in HIV-infected patients [2]. In addition to sepsis, HIV itself carries an independent risk for the development of AKI, and AKI in HIV-infected patients is often multifactorial [3]. While the risk of AKI is high in this population, developing sepsis increases their risk for the development of sepsis-associated AKI. Among patients admitted to hospital with sepsis in Uganda, about 57 − 82 % are living with HIV [1, 4, 5]. We, therefore, hypothesized that the incidence of AKI is higher in a population living with HIV presenting with sepsis than has been reported in studies that focus on either the incidence of AKI in HIV or in sepsis independently. The goal of our study was to determine the incidence and risk predictors of AKI among HIV-infected patients presenting with sepsis to the medical emergency department at Mbarara regional referral hospital in southwestern Uganda.

## Methods

### Study design, setting, and population

 We prospectively screened Ugandan adults at Mbarara Regional Referral Hospital (MRRH) which is located in Mbarara city, about 260 km from Kampala, the capital city of Uganda. MRRH serves as a teaching hospital for Mbarara University of Science and Technology and serves a population of approximately 8 million people in south-western Uganda. From March 2020 to July 2020, we enrolled patients who fit the inclusion criteria. Adult patients (≥ 18 years) presenting with sepsis were routinely screened for HIV and those who were positive were asked to provide informed consent before enrollment. The consent forms were translated into the local language for easy communication, and for those with altered mental status, we sought surrogate consent as per the declaration of Helsinki.

### Variables

In our study, individual patient data were collected. We were interested in sociodemographic characteristics like age, gender, level of education in years, duration of symptoms, ART regimen, and history of taking herbal or traditional medicine. The clinical variables included physical examination findings like blood pressure, respiratory rate, and Glasgow coma score to calculate the qSOFA. We also documented the mid upper arm circumference (MUAC), body temperature, and the commonly diagnosed opportunistic infections in HIV such as tuberculosis, cryptococcal meningitis, and pneumocystis pneumonia. Laboratory variables that were documented included an HIV test, complete blood count (CBC) (Sysmex XS 1000i) and differentials, baseline and 48-hour serum creatinine (as recommended by Kidney Disease Improving Global Outcome (KDIGO)) and urea (Humastar HS -200), as well as CD4 T cell count (PIMA Alere Inc., Waltham MA) at admission. The history of recently taking herbal medication was provided by the study patients or their caregivers, but for other prescription-medication taken, we used referral or past documents for reference.

**Fig. 1:**
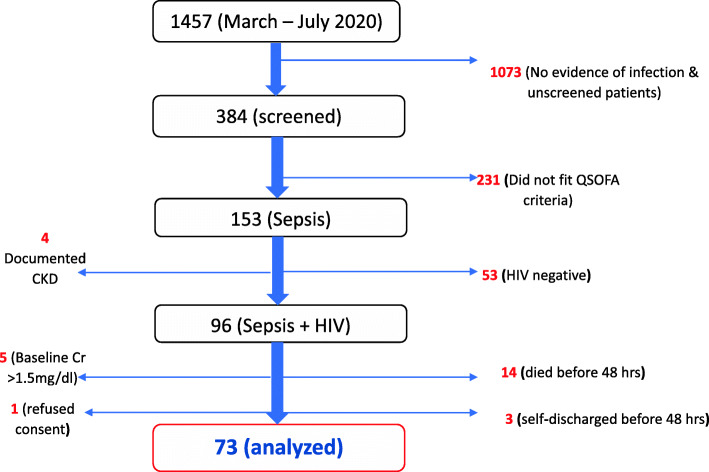
Flow chart showing participants’ recruitment

We used the variables collected to define sepsis using the qSOFA criteria. Patients with a score ≥ 2 in the presence of infection were included in our study[6]. Further inclusion criteria included age ≥ 18 years, documented HIV infection, and willingness to stay in hospital for at least 48 h. We excluded patients < 18 years, patients with a documented history of Chronic Kidney Disease (CKD), patients with a baseline creatinine > 1.5 mg/dl, and those with documented evidence of AKI at admission. AKI was defined as a rise in serum creatinine by 0.3 mg or more, after 48 h from the baseline measurement [7]. Data collection is still on-going as part of a bigger prospective study determining the long-term outcomes of patients with sepsis who develop AKI in a low resource setting. Details of participants’ recruitment are illustrated in Fig. 1.

### Statistical analysis

Data were entered into Microsoft Excel and exported to STATA version 15 (StataCorp, Lakeway Drive, College Station, Texas, USA) for analysis. Continuous variables were summarized by mean (SD) and skewed variables were summarized by median (IQR). We then calculated the cumulative incidence of AKI using the number of study participants who developed AKI after 48 h as the numerator out of the total number (denominator) of participants enrolled in the study, and a percentage was calculated. To determine independent risk predictors of AKI, multivariable binomial regressions were fitted and a *p*-value of ≤ 0.01 was considered statistically significant.

## Results

Among 73 patients enrolled from March to July 2020, the median age was 38 (interquartile range [IQR], 29–46 years), 44 patients (60.3 %) were women, and 39 patients (53.4 %) had been on ART for at least six or more months (Table 1**)**. At admission, 27 patients (37.0 %) had previously taken herbal medicine while 16 patients (21.9 %) had taken NSAIDs. Others had either taken Aminoglycosides (12 (16.4 %)) or Amphotericin B (4(5.5 %)). Among the vital signs, the median respiratory rate was 26 (IQR 24–32) cycles per minute, the median systolic blood pressure was 100 (90–103) mmHg, the median oxygen saturation was 93 (IQR 85–96) %, the median temperature was 38.0 (IQR 37.6–38.5) degrees centigrade, while the median pulse rate was 104 (IQR 83–120) beats per minute. The medium CD4 cell count among our participants was 67 (IQR 35–200), the median baseline creatinine was 0.93 (IQR 0.77–1.12) mg/dl, the median baseline urea was 26.8 (IQR 19.6–48.7) mg/dl, the median baseline white blood cell count was 4.52 (IQR 3.1–6.6) cells per microliter, the median hemoglobin was 10.5 (IQR 7.9–12.4) g/dl, while the median platelet count was 185 (IQR 128–304) cells/microliter.

**Table 1:** Baseline characteristics

Characteristic	*N *= 73, n (%)
Socio-demographics: n (%)	
Age in years, median (IQR)	38 (29–46)
Male gender, n (%)	44 (60.3)
Occupation, n (%) Employed Peasant farmer Unemployed	33 (45.2) 22 (30.1) 18 (24.7)
Level of education, n (%) None Primary Secondary and beyond	12 (16.4) 36 (49.3) 25 (34.3)
HAART duration categories, n (%) Not on HAART/Newly diagnosed < 6 months ≥ 6 months	17 (23.3) 17 (23.3) 39 (53.4)
Medications, n (%) TDF Herbs NSAIDS Aminoglycosides Amphotericin B	54 (74.0) 27 (37.0) 16 (21.9) 12 (16.4) 4 (5.48)
Clinical characteristics	
Focus of sepsis, n (%) Unknown Respiratory system CNS Others (Gastrointestinal, hematologic)	26 (35.6) 24 (32.9) 16 (21.9) 7 (9.6)
MUAC in cm, median (IQR)	23 (20–27)
Respiratory rate in cycles/min, median (IQR)	26 (24–32)
Systolic BP in mmHg, median (IQR)	100 (90–103)
Oxygen saturation in %, median (IQR)	93 (85–96)
Temperature in °C, median (IQR)	38 (37.6–38.5)
Pulse rate in bpm, median (IQR)	104 (83–120)
CD4 count in cells/ul, median (IQR)	6700 (3500–20,000)
Baseline creatinine in mg/dl, median (IQR)	0.93 (0.77–1.12)
Baseline urea in mg/dl, median (IQR)	26.8 (19.6–48.7)
WBC count in cells/ul, median (IQR)	4.52 (3.1–6.6)
Hemoglobin in g/dl, median (IQR)	10.5 (7.9–12.4)
Platelet count in cells/ul, median (IQR)	185 (128–304)

Abbreviations: *HAART* Highly Active Anti-Retroviral Therapy, *TDF* Tenofovir, *NSAIDS* Non-steroidal anti-inflammatory drugs, *CNS* Central Nervous System, *MUAC* Mid upper arm circumference, *BP* Blood pressure, *CD* Cluster Differentiation, *WBC* White blood cell

The cumulative incidence of AKI in 48 h post-admission was 19.2 %.

On multivariate analysis, the presence of thrombocytopenia (platelet count less than 150) (RR 8.21, 95 % CI: 2.0–33.8, *p* = 0.004) was significantly associated with AKI. The details of the analysis are shown in Table 2.

**Table 2:** Crude and Adjusted analysis

Characteristic	Crude analysis cRR (95 % CI)	*P* value	Adjusted analysis ARR (95 % CI)	*P* value
Male gender	1.65 (0.57–4.76)	0.356	1.4 (0.53–3.69)	0.491
**MUAC** < 24 cm ≥ 24 cm	0.73 (0.28–1.89) Ref	0.517	0.90 (0.39–2.09)	0.813
**CD4 count** < 200 ≥ 200	0.63 (0.24–1.65) Ref	0.351	0.53 (0.21–1.31)	0.168
**WBC Count** **≤** 12,000 > 12,000	Ref 1.33 (0.23–7.76)	0.754	3.69 (0.38–35.49)	0.258
**Systolic BP** < 100 ≥ 100	1.15 (0.45–2.94) Ref	0.775	1.22 (0.48–3.12)	0.680
**Platelet count** < 150 ≥ 150	6.27 (1.91–20.43) Ref	0.002	8.21 (2.00–33.80)	**0.004**

Abbreviations: *MUAC* Mid upper arm circumference, *CD* Cluster differentiation *WBC* White blood cell, *BP* Blood pressure

## Discussion

 In this prospective study, our main goal was to establish the incidence and risk predictors of AKI among HIV-infected adults admitted to the medical emergency ward of Mbarara Regional Referral Hospital (MRRH). We found a high cumulative incidence of AKI at 19.2 %, and thrombocytopenia as the main independent risk predictor of AKI, within the first 48 h after admission. To the best of our knowledge, this is the first study done in an HIV-endemic area to address the incidence of AKI among HIV-positive patients admitted with sepsis.

In our study, the incidence of AKI among HIV-positive patients admitted with sepsis was 19.2 % which is higher than that found by Hsu et al. at the emergency department of a university hospital in Taiwan, where the incidence of AKI among patients with sepsis was 14.2 % [8]. In Hsu’s study, however, there were no HIV-positive patients, and those with CKD were included. This study did not include HIV-positive patients. The only study that addressed the burden of AKI in sepsis in Sub-Saharan Africa (SSA) and where the majority of the participants were HIV-positive, was a hospital-based cross-sectional study done in Uganda by Bagasha and colleagues [1]. This study found a prevalence of AKI of 16.3 % of participants with sepsis admitted to medical wards [1].

In high resource settings, however, the incidence of AKI seems to be higher than what we found in our study. A study done in Japan by Medeiros and colleagues at a tertiary hospital found an incidence of AKI of 72 % among patients with sepsis [9]. A retrospective single-center study done at an emergency department in South Korea by Suh et al. found an incidence of 57.7 % among patients with sepsis [10]. Two other studies, by Bagshaw et al. and Lopes et al., were both done in an ICU setting and found an incidence of 42 and 37.4 % respectively [11, 12]. Since this study was done in an ICU, the disease severity may have contributed to the higher incidence rates of AKI among these patients. Whereas the incidence of AKI was high, the studies done by Bagshaw, Suh, and Medeiros included patients with CKD, and generally, the patients had a higher mean age than our study. Advanced age and underlying renal dysfunction lower the renal reserve, which is likely to influence the higher rates of development of AKI [13, 14].

Thrombocytopenia was significantly associated with AKI in HIV-positive patients admitted with sepsis. This is a similar finding in the study done in South Korea by Suh and colleagues which also showed thrombocytopenia as an independent risk factor for AKI in patients with sepsis [10].

The association of thrombocytopenia and AKI is likely due to; the pattern of serum cytokine levels due to sepsis that is associated with prolonged thrombocytopenia [15], therefore lower platelet counts might indicate more severe immunologic responses subsequently leading to AKI. Also, thrombotic microangiopathies have been found in up to 7 % of HIV-infected patients [16], due to the cytotoxic effects of HIV or opportunistic infections [17]. Low platelets may, therefore, be a marker of severe thrombotic microangiopathies due to HIV infection, which is also an independent risk factor for AKI even in the absence of sepsis [18].

 To our knowledge, this is the first peer-reviewed study from SSA investigating the burden and risk of AKI in HIV-infected patients presenting with sepsis. We were limited by the lack of novel tests for AKI such as Cystatin-C and Neutrophil Gelatinase-associated Lipocalin (NGAL). Creatinine delays to rise in AKI which would not make it an ideal marker for use in early detection of AKI. More so, the majority of our patients died before the 48-hour mark for a second creatinine measurement, yet they could have had the outcome of interest.

## Conclusions

There is a high incidence of early AKI among HIV-positive patients admitted with sepsis. Thrombocytopenia at admission is significantly associated with the development of AKI within 48 h after hospitalization. We recommend a larger prospective study that will include point of care blood cultures, biomarkers of sepsis and kidney injury, and long-term follow-up of patients with HIV presenting with sepsis.

## Data Availability

The datasets used during the current study are available from the corresponding author on reasonable request.

## Abbreviations

AKI: Acute Kidney Injury
ART: Antiretroviral Therapy
CKD: Chronic Kidney Disease
KDIGO: Kidney Disease Improving Global Outcome
MRRH: Mbarara Regional Referral Hospital
MUAC: Mid Upper Arm Circumference
qSOFA: Quick Sequential Organ Failure Assessment
